# Critiquing Construct Validity in World City Network Research: Moving from Office Location Networks to Inter‐Organizational Projects in the Modeling of Intercity Business Flows

**DOI:** 10.1111/gean.12226

**Published:** 2019-12-19

**Authors:** Vladimír Pažitka, Dariusz Wójcik, Eric Knight

**Affiliations:** ^1^ School of Geography and the Environment Oxford University Oxford UK; ^2^ University of Sydney Business School University of Sydney Sydney Australia

## Abstract

The interlocking world city network model and other office location approaches (OLAs) have become the most widely used empirical models of the world city network (WCN). Despite numerous methodological improvements, they continue to rely on a legacy of using data on office locations of firms to indirectly estimate intercity business flows. To advance the dialogue about how to improve on existing empirical models of the WCN, we examine the content, construct and structural validity of OLAs. We analyze the link between the theoretical construct of intercity business flows and network projections obtained from office location data and uncover evidence that calls into question the validity of OLAs as empirical models of the WCN. In the spirit of no deconstruction without reconstruction, we then develop an alternative empirical model of the WCN, based on directly observable relational ties among APS firms, which are formed through co‐production of complex services. We call this the inter‐organizational project approach (IOPA). We argue for IOPA's construct validity as an empirical model of the WCN and offer empirical evidence for its structural validity. We demonstrate it using a global sample of 161,114 investment bank syndicates in the 2000–2015 period.

## Introduction

We have to deploy the strategy of employing indirect measures because measuring actual business flows in our research on global inter‐city relations is simply impossible (Taylor and Derudder 2016, 38).

Since Taylor's (2001) specification of the interlocking world city network model (IWCNM), it has become the most widely used empirical model of the world city network (WCN). IWCNM and its various derivatives, referred to as office‐location approaches (OLAs), have generated a substantial empirical literature on a variety of advanced producer services (APS) industries including law, accountancy, marketing, management consultancy, and financial services (Taylor and Derudder 2016). The initial research agenda of the Globalization and World Cities (GaWC) research group was to overcome the “dirty little secret” of the global and world city research, identified as the lack of data and empirical evidence on intercity network connections associated with the activities of APS firms (Short et al. 1996, 697). The IWCNM has been proposed and accepted by many as an empirically feasible approach to addressing this gap. Although OLAs have been methodologically refined since, they still carry the legacy of APS firms' office location data as the primary data input into these models, and the basis for indirect estimation of intercity business flows (Neal 2014; Taylor and Derudder 2016). As new data sources become available, we believe that this presents a major opportunity to reconsider the reliance of WCN research on untested assumptions related to the ability of network projections from office location data to validly represent intercity business flows.

In order to contribute to this debate, our first objective is to examine the content, construct and structural validity of OLAs as empirical models of the WCN. This exercise involves an examination of the link between the underlying theoretical construct of intercity business flows, which represent the ties among cities in the WCN (Taylor and Derudder 2016), and the IWCNM as its most widely used empirical model and the methodological foundation of other OLAs. We follow the work of Cronbach and Meehl (1955) and Messick (1995) to structure our examination of content and construct validity and consider how IWCNM is designed to empirically identify variation in the underlying intercity business flows. We also discuss the potential sources of variation in projections of intercity business flows obtained using IWCNM, which may not relate to the underlying intercity business flows (Messick 1995). We complement our argument with empirical evidence that tests the structural validity of IWCNM and other OLAs by correlating their projected intercity business flows with variables that relate to resistance to intercity business flows and potential for intercity business flows.

The second objective of this article is to develop, formalize and validate an empirical model of the WCN, based on directly observable relational ties among APS firms, formed through inter‐organizational projects, which are directly related to co‐production of complex services. We base this approach on inter‐organizational project participation of APS firms, which relates directly to the co‐production of the services they provide to their clients. Network ties formed through such inter‐organizational projects are then intrinsically linked to the business flows among participating APS firms. In doing so, we address criticism faced by the WCN literature, related to the inability of IWCNM to test the core hypothesis of global city theory—the co‐production of complex services within advanced producer services (APS) complexes (Smith and Doel 2011). The inter‐organizational project approach (IOPA) to modeling urban networks developed here is compatible with the existing WCN theory, particularly the theoretical constructs of intercity business flows (Taylor and Derudder 2016), global city (Sassen 2001) and urban networks formed by social interactions (Castells 1996). Instead of relying on network projections from data on office locations of APS firms as an indirect proxy, we argue that network projections based on data on inter‐organizational projects more directly capture business flows among APS firms. Crucially, many of the ties formed through inter‐organizational projects span the boundaries of cities and consequently form an urban network of intercity business flows, as envisioned by Castells (1996), Sassen (2002) and foregrounded in a growing body of work linking economic geography with corporate strategy (Knight and Wójcik 2017). We argue that our IOPA model fills numerous conceptually important gaps identified by IWCNM’s critics and allows for an empirical analysis of co‐production of complex services by APS firms (Smith and Doel 2011) and firm‐level strategic activities across networks (Knight and Wójcik 2017).

Our third objective is to use IOPA as a reference model for testing the convergent validity of OLAs. We accomplish this task using the framework for assessing validity of empirical measures developed by Cronbach and Meehl (1955) and Messick (1995). We first provide an empirical demonstration of IOPA using data on 161,114 underwriting syndicates for the 2000–2015 period. We then compare the IOPA network structures with those of OLAs using data on inter‐organizational projects and office locations of our sampled firms for 2015. In doing so, we empirically test the assumption that IWCNM and other OLAs produce projections of the WCN that are representative of actual intercity business flows.

In the next section, we review the state of the art in the WCN research and discuss some of the core assumptions and limitations underlying the IWCNM methodology. We see these assumptions and limitations regarding the use of office location data in studying intercity business flows and the structure of the WCN as being relevant to IWCNM's construct validity as well as that of other OLAs, which share the same methodological foundations. As part of this section, we also explore other strands of research and competing paradigms to modeling urban networks and draw on these works in developing the IOPA. We formalize the IOPA in the following section and argue that it offers a theory consistent empirical representation of the WCN, thus building a case for its construct validity. We then outline our methodology for conducting tests of structural validity and comparing the network structures of different modeling approaches, which we later utilize to conduct tests of convergent validity. In the results section, we offer an empirical illustration of IOPA and present the results of our tests of convergent and structural validity. In the concluding section, we reflect on the implications of our findings regarding OLAs' validity as empirical models of the WCN and discuss the opportunities and limitations in using IOPA as an alternative empirical model of the WCN.

## The state of the art in world city network research

The WCN concept and IWCNM, as its most widely used empirical model, are rooted in the theory of the global city (Sassen 1991) and Castells' (1996) conceptualization of cities as spaces of flows. Sassen (1991) considers the role of APS in urban development and proposes that concentration of accountancy, advertising, management consultancy and financial services firms in the business districts of leading metropolitan areas is driven by the co‐production of APS and the associated need for proximity among different types of APS firms. This process is then shown to have broader consequences for urban development, inequality, spatial organization of industries beyond APS and the experience of women living in urban areas (Sassen 2011).

While Friedmann's (1986) world city hypothesis is an important precursor to the contemporary WCN literature, Sassen's (1991) global city signifies a shift in focus from corporate headquarters and their control over parts of the economy to the notion of APS as services that allow multinational enterprises (MNEs) to coordinate their spatially dispersed, often global, business activities to enact corporate strategy (Knight and Wójcik 2017). Castells (1996) extends Sassen's (1991) global city concept to a global network of cities, which are defined by social interactions and practices that flow through them, a position later adopted by Sassen herself. “[T]here is […] no such entity as a single global city […], the category ‘global city’ only makes sense as a component of a global network of strategic sites” (Sassen 2002, 31).

Consequently, the focus of the global and world city research has shifted from the co‐production of APS at the level of individual cities to a WCN formed by interactions among APS firms (Taylor 2004). Such interactions are termed *intercity business flows* in related research. They have become the elusive object of study of the substantial empirical literature that follows Taylor's (2001) seminal paper that introduces the IWCNM as an empirically feasible model of the WCN. Until the formation of the GaWC research group, there had been little empirical evidence on intercity business flows facilitated by APS firms (Short et al. 1996). The lack of suitable data sources to study such flows directly led Taylor (2001) to resort to an indirect measure of intercity business flows—a bipartite projection of a firm–city affiliation matrix constructed using data on office locations of APS firms. This is operationalized by using a matrix of service values *V*, with rows representing cities and columns representing APS firms. Elements of *V* ‐ *v_ij_* are customarily coded on a 0 to 5 scale, with 0 for non‐presence in a city and 5 for a global head office. The individual network connectivity scores *r_ij_* representing the strength of connection between two offices of a given firm are calculated as the product of their service values (equation 1). Estimates of business flows for city‐dyads are calculated as the sums of connectivity scores across their overlapping firms (equation 2). Finally, to calculate a city’s network centrality *N_a_*, Taylor (2001) sums up the relational elements *r_ij_* for each city (equation 3).

(1) $$r_{ab,j}=v_{\mathrm{aj}}\cdot v_{\mathrm{bj}}$$

(2) $$r_{\mathrm{ab}}=\sum_{j}r_{ab,j}$$

(3) $$N_{a}=\sum_{i}r_{\mathrm{ai}};\;a\neq i$$

Thus, these projections of intercity business flows are based on attribute data on the codified importance and location of offices (Nordlund 2004). As a result, any empirical research based on this specification of intercity business flows assumes that such projections are representative of the network structure of actual intercity business flows. This reasoning entails the following assumptions:

1. The volume of intercity business flows is associated with the number of overlapping firms with offices located across a given city‐dyad and the relative importance of these offices.

This means that the offices of firms, one of the material supports for intercity business flows, are treated as an indicator of social interactions, their volume and structure. Although it is plausible that the presence of offices of the same firms across a city‐dyad helps to facilitate interactions among cities, it is unclear how precisely intercity business flows are related to such office networks and what is the explanatory power of office networks in explaining intercity business flows. The use of office location data to project intercity business flows therefore becomes particularly problematic in the contemporary world of business, where firm actors are increasingly mobile and the internet and platform economies enable offshore deal‐making regardless of physical office presence (Knight and Wójcik 2017).

- Intercity business flows are aggregates of intra‐firm business flows among offices located across a city‐dyad.

Due to this assumption, which is implicit in IWCNM’s functional form, we argue that interfirm business flows are not adequately accounted for in this urban network structure. This is problematic as is the assumption that single location firms do not contribute to intercity business flows. Ultimately, this leads to a potentially large fraction of the variation in intercity business flows being omitted from IWCNM’s projections.

- Intra‐firm business flows only vary with the relative size and location of offices involved and are otherwise the same across different firms and industries.

This assumption imposes strong regularity conditions on the propensity of offices of different firms, potentially operating in different industries, to interact with each other. It means that offices of accounting, law, marketing, financial services and management consultancy firms are all just as likely to interact with other offices within their firms, without any variation due to industry or firm‐specific business practices. Consequently, variation in the level of business flows due to firm‐specific and industry‐related practices is omitted in projections of intercity business flows derived using IWCNM.

Notwithstanding these assumptions, IWCNM and OLAs have become the most widely used empirical models of the WCN, given their ability to offer empirical evidence, albeit indirect, based on publicly available data from corporate websites (Taylor and Derudder 2016) and financial databases (Sigler and Martinus 2016). Empirical contributions building on the IWCNM methodology include studies of banking networks (Rossi, Beaverstock, and Taylor 2007), NGOs and UN agencies (Taylor 2005), publicly listed corporations (Sigler and Martinus 2016), APS firms (Taylor et al. 2014), evolution of urban networks (Liu et al. 2014) and contrasting the role of APS firms and cities in the WCN (Taylor et al. 2014).

Since its conception, IWCNM has attracted a substantial interest as well as constructive criticism related to its methodology and the theory underpinning the global and world city research (Robinson 2002, 2005, 2006; Smith 2003a, b, 2014; Nordlund 2004; Neal 2012, 2013, 2014, 2016). Taylor's (2001) legacy of using network projections from office location data as an indirect measure of intercity business flows is viewed as a key limitation, given that the intercity business flows among APS firms' offices need to be assumed rather than directly observed (Nordlund 2004). Naturally, the problem here is not the plausibility of the assumption that such flows exist among offices of firms, but rather that the network structure derived from office location data is representative of the structure of actual intercity business flows (Neal 2012). Nordlund (2004) points out that the projection function applied by Taylor (2001) to obtain city adjacency matrix converts attribute data on office locations to relational data on intercity business flows. Although it is possible mathematically, the validity of this operation relies on assumptions 1–3 outlined earlier in this section. Neal (2012) elaborates on Nordlund's (2004) critique and shows that network structures produced by IWCNM are subject to structurally deterministic biases that compromise their empirical validity for assessing network density, cliquishness and render centrality measures that rely on the structure of the entire network unreliable. Upon acknowledging that these problems stem from the use of attribute data on office locations to derive projections of intercity network connections, Neal (2012) calls for the use of relational data on interactions of APS firms, to rectify these crucial deficiencies of the IWCNM methodology.

Another group of methodological critiques deals with the problem of interpreting and assessing the measures of network centrality of cities. Rather than interpreting network centralities at their face value, comparison with a null model is proposed (Neal 2013). Neal (2016) addresses this by adapting the stochastic degree sequence model to detect city‐dyads, which are more strongly connected than would have been expected, if APS firms' offices drawn from the same degree distribution were randomly assigned to cities. Hennemann and Derudder (2014) purse a similar exercise using a randomized baseline model. The common goal of these studies is to identify city‐dyads, which are likely to be connected due to a strategic firm level activity, rather than random chance.

Robinson (2002) critiques the global and world city research for being narrowly focused on APS firms and cities in the global north. As a result, not only a potentially more nuanced narrative of urban development and urban network connectivity is missed, but the successful cities of the global north may be uncritically adopted as effective role models for the struggling cities of the global south. Arguably, the risk of an uncritical adoption of a particular model of urban development by policymakers becomes a problem, if the specific context of each city is not taken into account (Robinson 2005, 2006). While Robinson’s critique is mainly focused on the scope of global and world city research, Smith (2003a, b) challenges the theoretical foundations and methods of the WCN research based on the works of Friedmann (1986), Sassen (1991, 2001), Castells (1996) and Taylor (2001). The essence of Smith's (2003a, b) critique relates to the focus of global and world city research on a limited set of phenomena and presupposed organizing principles, which drive globalization and WCN formation, including the activities of APS firms, technological development and processes of capitalist accumulation. This means that non‐economic social interactions, human agency and contingency that shape and form the WCN are not given appropriate consideration (Smith 2003a). In order to overcome these limitations, actor‐network theory and non‐representational theory are offered as suitable alternatives (Smith 2003a, b). Being rooted in poststructuralism, these theoretical frameworks are however incompatible with quantitative analytical methods and instead are designed with qualitative research in mind (Smith 2003a).

In a follow‐up article, Smith (2014) challenges the notion of *command and control* emanating from world cities as one of the organizing principles of the global economy. In response, Bassens and van Meeteren (2015), following Wójcik (2013), argue that although there is no absolute command and control, there is a deeply rooted logic of financialization and APS firms occupy a central position in decision‐making networks that shape the global economy. The perceived lack of conceptual progress in the WCN research in recent years has been linked to the disproportionate focus of researchers on quantitative studies, primarily relying on OLAs, which may have in their present form effectively exhausted their potential to drive this field forward (Watson and Beaverstock 2014). While we agree that rigorous qualitative research is needed to aid theory building, there is also a valid need to develop quantitative methods that allow for an improved representation of the existing theoretical constructs. It is essential for researchers interested in WCN to be able to empirically test theoretical propositions relating to the co‐production of services (Sassen 2001) and to be able to explore the structure of intercity business flows that form the WCN (Taylor and Derudder 2016). For this reason, we now turn to the link between presently used empirical measures and theoretical constructs in the WCN research.

Notwithstanding lively debates surrounding theories and methods in the WCN research, discussions about construct validity (Cronbach and Meehl 1955), the presence of a reliable link between empirical measures and theoretical constructs, have been surprisingly rare. Smith and Doel (2011) are an exception in this respect and identify a disconnect between IWCNM methodology and the global city theory. They argue that IWCNM is unable to test claims related to the co‐production of services within APS complexes, a central tenet of the global city theory (Sassen 2001). Similarly, IWCNM and other OLAs cannot be used to study the co‐production of APS across a network of global cities (Castells 1996; Sassen 2002). Coupled with the three assumptions discussed earlier, which relate to the structure of the WCN projected by IWCNM, this apparent disconnect between the theoretical core of the global and world city research and IWCNM raises important concerns about the construct validity of IWCNM and other OLAs.

Neal (2014) also considers the problem of construct validity^1^ in WCN research and argues for potential validity of OLAs including IWCNM (Taylor 2001), sorting (Neal 2013), normalized Bonacich (1972) and naïve model (Neal 2014). Such potential validity relies on asserted links among different OLAs and different conceptualizations of the WCN, which Neal (2014) derives from the works of various authors including Robinson (2002) and Smith (2014). The conclusions drawn by Neal (2014) deserve critical attention in our view as they assert validity of empirical models as representations of theoretical constructs without offering a critical discussion of whether such a link could plausibly hold.

As an example, Robinson (2002) is critical of the narrow scope of global and world city research and its focus on APS firms. Instead, she develops her own concept of *ordinary city* and calls for a more nuanced and context‐dependent approach to urban development, which avoids prescribing a single model for all cities (Robinson 2006). However, Neal (2014, 433) asserts that “[f]or theorists like Robinson (2002) […], who think of the world city network as something not related to cities’ sizes, normalized projections offer a valid measure.” In the same article, Neal (2014) defines the size‐normalized model of the WCN as a projection from a matrix of office locations of APS firms. It certainly raises concerns about whether this model could be reconciled with Robinson's (2002) theorizing of cities.

In a similar fashion, Neal (2014) asserts that the sorting model developed by Neal (2013), which is based on the statistical significance of links in an underlying IWCNM network projection, is a suitable representation for Smith's (2014) conceptualization of the WCN. However, we would argue that OLAs are simply not compatible with Smith's (2014) poststructuralist approach to urban studies, which is highly critical of quantitative models of urban networks. Similarly, the disconnect between the key concepts of the global city theory, namely the co‐production of APS within global cities, and what is in fact measured using OLAs, identified by Smith and Doel (2011), is not addressed by Neal (2014) in his discussion of construct validity of OLAs. Neither are empirical tests conducted to evaluate the structural validity of OLAs’ network structures or their convergent validity. The latter point may be attributed to the lack of a suitable reference model, which has hindered this line of research in the past. Given these challenges encountered by previous studies on the link between the theoretical construct of the WCN and its presently available empirical models, we understand that in addition to scrutinizing the construct and structural validity of these models, it is essential to explore other potential empirical models that could represent the WCN, as specified by Taylor and Derudder (2016).

Although the IWCNM and OLAs are the most popular empirical models of the WCN, alternative approaches for studying urban networks are now emerging, based on alternative and increasingly granular data. These can be broadly divided into (1) infrastructure, (2) corporate organization, (3) labor mobility and (4) transactional approaches. Infrastructure approaches focus on physical transportation and telecommunication networks, but they are conceptually weakly connected to the WCN theory (Smith and Timberlake 2001; Taylor and Derudder 2016). Corporate organization studies are based on parent–subsidiary ownership links of MNEs and span a wide range of industries (Alderson and Beckfield 2004). However, these studies are based on Friedmann's (1986) world city concept and therefore cannot be readily applied to the analysis of WCN, based on global city theory (Taylor and Derudder 2016). Beaverstock (2007) develops an approach based on labor market mobility in the global investment banking industry, which allows for an examination of both micro and macro networks formed by flows created by intercity business trips, fixed term assignments, as well as labor force mobility across different organizations. While this approach provides a novel and nuanced approach to directly studying specific types of intercity business flows, it remains a substantial empirical challenge to extend this approach to a large‐scale global analysis and to obtain representative sample sizes.

Transactional studies include the work of Rossi, Beaverstock, and Taylor (2007) and Hanssens, Derudder, and Witlox (2012), who pursue an approach based on APS firm–client transactions, surveying how companies engage with their APS providers. Pan et al. (2017) use data on Chinese A‐share IPOs to study urban networks through the interactions of accounting, securities and law firms. In a related study, Pan et al. (2018) investigate the geography of underwriter–client ties in Chinese IPOs. Van Meeteren and Bassens (2016) offer a detailed analysis of three Eurobond issues and illuminate the complexity of networks of APS firms involved in these transactions. This body of work shows that ties among APS firms that cross the boundaries of metropolitan areas are commonplace. At the same time, such approaches are scalable and allow for a large sample global analysis, which can be representative of a truly global urban network.

To advance this literature, we believe that it is necessary to further develop, formalize and most importantly validate empirical models of the WCN. Suitable empirical models should meet the criteria for construct validity (Cronbach and Meehl 1955), meaning that the network structures that they produce should be representative of the structure of the underlying intercity business flows, which represent the ties among cities in the WCN (Taylor and Derudder 2016). The first step of such an endeavor is to identify suitable empirical building blocks of the WCN. Such building blocks should ideally be directly observable relational ties among the underlying actors, individual APS firms, and should be as directly as possible linked to the business flows among them. These building blocks could then serve as the primary data input into models of urban networks, providing an alternative to office locations of firms, which serve this function in much of the existing WCN research (Taylor and Derudder 2016). Recent research proposes ties among accounting, law and securities firms working on the same IPOs (Pan et al. 2017) and ties among APS firms and their clients (Rossi, Beaverstock, and Taylor 2007; Hanssens, Derudder, and Witlox 2012; Pan et al. 2018) as potential empirical building blocks of the WCN. We build on these ideas in the next section, to develop, formalize and validate the IOPA.

## Inter‐organizational projects as the empirical building blocks of the world city network

In contrast to OLAs' reliance on office location data, the IOPA developed here uses inter‐organizational projects as the empirical building blocks of the WCN. We operationalize this using underwriting syndicates for primary issues of equity and debt securities. These transactions are indispensable for the functioning of capital markets and allow issuers to raise financial capital from investors. Investment banks serve as underwriters of these transactions and intermediate market interactions among issuers and investors (Wójcik et al. 2018). We present records of membership in underwriting syndicates in the form a bank–deal affiliation matrix *D*. The elements of matrix *D* ‐ *d_ij_* denote the participation of banks in underwriting syndicates (deals) and can be either binary or weighted by revenue earned. To obtain an adjacency matrix of bank connectivity *B*, we apply a projection function shown in equation (4).

(4) $$B=D\left(D^{T}\right)\;;\;b_{\mathrm{ij}}=0\;\text{if}\;i=j$$

where *D* is a bank–deal affiliation matrix, *D^T^* is a transpose of *D*, and *B* is a weighted bank adjacency matrix. Depending on the choice of weights in *D*, the elements of *B* ‐ *b_ij_* are interpreted as either frequencies of co‐membership of bank‐dyads in syndicated deals (*d_ij_* are binary) or as value weighted business flows among banks (*d_ij_* are revenues from each deal apportioned by bank).

To obtain intercity ties, we proceed by converting the bank adjacency matrix *B* into an edge‐list of bank‐dyads and include frequency of syndication as a weight for each edge *b_ij_*. We add the city of operational headquarters to every bank's subsidiary in this edge list. We then sum the bank‐bank edge weights *b_ij,vw_* by city‐dyads *vw*, thus converting the original bank level edge‐list to a city level edge‐list (equation 5). Finally, we convert it to a weighted city adjacency matrix *C* with elements *c_vw_*, which represent intercity business flows formed by co‐syndication of banks' subsidiaries located in cities *v* and *w*.

(5) $$c_{\mathrm{vw}}=\sum_{\mathrm{vw}}b_{ij,vw}$$

For a binary bank–deal affiliation matrix, the elements of matrix *C* (referred to as IOPA (count)) ‐ *c_vw_* are constructed as frequencies of syndication ties among banks' subsidiaries located in cities *v* and *w*. Alternatively, for a revenue weighted bank–deal affiliation matrix *D*, elements of matrix *C* (referred to as IOPA (revenue)) ‐ *c_vw_* are revenue weighted syndication ties among banks' subsidiaries located in cities *v* and *w*.

We use the group degree centrality to calculate city network centrality scores (Everett and Borgatti 1999). Group degree centrality is based on the notion that only non‐redundant connections among group members add to its centrality. We therefore count the number of banks outside city *a*, to which at least one bank within city *a* is connected. To make these measures comparable across time, networks of varying sizes, and most importantly across different cities, we normalize each centrality score by its maximum possible value (Everett and Borgatti 1999).

### Construct validity

Given our intention to use IOPA as a reference model for assessing the convergent validity of OLAs, we must first establish that IOPA is a valid empirical model of the WCN. In the absence of another widely accepted and known to be valid empirical model of WCN, we escape the circular problem of comparing each model to another, more valid model by arguing for the construct validity of IOPA. We do this by establishing a link between the relational ties formed among firms through inter‐organizational projects and the theoretical construct of intercity business flows that IOPA aims to measure (Cronbach and Meehl 1955). Only once such reasoning can be accepted, does it make sense to use it as a reference model, to empirically evaluate the convergent validity of OLAs. We present our argument by examining OLAs and IOPA in turns to evaluate how well each of these approaches meets the criteria for content and construct validity (Cronbach and Meehl 1955), before we move to the tests of structural validity (Messick 1995).

Content validity, a prerequisite for construct validity, can be established by connecting observable attributes of the proposed measure to the theoretical universe of interest (Cronbach and Meehl 1955). In the context of the WCN, this means linking observables such as records of emails, telephone calls, meetings, business travel, office locations, inter‐organizational projects or monetary flows to the theoretical construct of intercity business flows that form the WCN (Taylor and Derudder 2016). OLAs are based on firm–city affiliation matrices representing spatial organization of firms across cities. Taylor and Derudder (2016) propose that global networks of offices can represent the structure of intercity business flows, because such flows are expected to materialize among offices of APS firms located in different cities. The validity of this indirect approach relies on the representativeness of projections from a bipartite firm–city network to a one‐mode city network, which crucially relies on the three assumptions discussed in the previous section. In this article, we propose instead a link between intercity business flows and projections of the WCN derived from records of participation of APS firms in inter‐organizational projects. Records of participation in inter‐organizational projects are perhaps the most widely available relational data on the co‐production of APS as well as business flows among firms, which are at the heart of the WCN concept rooted in the global city theory (Sassen 2011). Ties among APS firms formed through inter‐organizational projects relating to the co‐production of services are directly linked to business flows among APS firms and across cities. Naturally, no single type of inter‐organizational project offers an unbiased and complete representation of the WCN. Instead it sheds light on one of its many facets. Both IOPA and OLAs appear to pass this first hurdle, as they can be plausibly linked to intercity business flows.

Arguing for construct validity requires establishing a link between empirical measure and a theoretical construct, which cannot be observed directly or in its totality (Cronbach and Meehl 1955). This requires researchers to identify what constructs account for variance in a given empirical measure. Provided intercity business flows within WCN are either difficult to measure directly or too complex for any empirical measure to represent fully, we must resort to empirical measures which are either indirect, incomplete, or both. Using projections of potential intercity business flows from firm–city affiliation matrices is one such indirect and incomplete measure (Taylor and Derudder 2016). In contrast, ties among APS firms formed through inter‐organizational projects are directly linked to the underlying business flows associated with such projects, although they are certainly an incomplete measure. To validate either of these measures of intercity business flows, one must show that (a) it varies in response to changes in the underlying intercity business flows and (b) only variation in intercity business flows drives the variation in the network structure obtained using a given measure. The second point relates to potential sources of spurious variation in empirical measures, which do not relate to the underlying construct being measured.

As we have argued in the literature review section, the structural properties that IWCNM imposes on urban network structures it generates (assumptions 1–3) are potential sources of construct invalidity. Although IWCNM’s projected intercity business flows are likely to be related to actual intercity business flows, there are sources of variation in intercity business flows that are unaccounted for by IWCNM, including interfirm interactions and differences across industries and firms in their propensity to interact at distance. There are also sources of spurious variation in IWCNM projections of intercity business flows that are not necessarily caused by variation in the underlying intercity business flows and are instead generated mechanically by IWCNM's methodological design. IWCNM predicts a specific pattern of potential intercity business flows derived by multiplying codified office sizes and summing them for each city‐dyad. Effectively, IWCNM presupposes that a material support for business flows, offices of firms, relate in a very specific and knowable manner to intercity business flows. However, there is no empirical evidence to support the validity of this assumption.

In contrast, IOPA relies on data on inter‐organizational projects, which allows us to directly observe relational ties among APS firms formed through co‐production of complex services. This means that the network structure it generates varies only when business flows associated with such projects materialize and they can be quantified either using frequencies of collaboration among firms or can be weighted by a revenue earned from such projects. This means that the observed network structure that IOPA generates is not driven by assumptions about corporate strategies, differences in business practices across industries or individual firms, and instead allows observed interactions among firms to illuminate the WCN structure. Consequently, IOPA meets the criteria of construct validity more adequately than IWCNM and other OLAs.

### Tests of structural validity

To provide empirical support for our argument, we conduct tests of structural validity of the most widely used OLAs and IOPA network structures (Messick 1995). The structural validity of both IOPA and OLAs can be evaluated independently of each other by examining their correlation with other variables that are believed to be associated with intercity business flows. Our test of structural validity is based on the following three axiom‐like tendencies widely documented in urban and economic geography (see e.g., Polèse 2010) as well as international economics (Moshirian, Li, and Sim 2005). First, intercity business flows decline on average with geographical distance. Second, intercity business flows are on average lower for city‐dyads separated by political borders. Third, intercity business flows are on average higher for dyads of large cities^2^ than small cities. Any inconsistencies with these tendencies can be interpreted as evidence of a structural invalidity. Additionally, we would expect the explanatory power (R^2^) of a model with these three explanatory variables to be decisively larger than zero, for a structurally valid dependent variable representing WCN structure.

To operationalize our tests of structural validity, we estimate a series of multivariate quadratic assignment procedure (QAP) regression models, one for each empirical model of the WCN. QAP regression is effectively the equivalent of a standard linear regression for network data and allows network structures, such as those produced by IOPA and OLAs to be used as dependent variables (Krackhardt 1988). We model the network structure produced by each OLA as well as IOPA as a function of geographical distances among cities, binary indicators for political borders and dyadic products of city sizes, a proxy for interaction potential among cities. To estimate our models in the form shown in equation (6), we apply the QAP multivariate regression estimator (Krackhardt 1988).

(6) $$y=\beta_{0}+\beta_{1}CbTies+\beta_{2}GeoDistance+\beta_{3}NaiveRevenue+\varepsilon$$

where y is a vector of edge weights obtained by vectorizing the adjacency matrix representing WCN derived using either IOPA or OLAs, *CbTies* is a vector of binary (0,1) indicators representing political borders, *GeoDistance* is a vector of geographical distances among cities, *NaiveRevenue* is a product of city sizes, measured by underwriting revenue, and serves as a proxy for interaction potential and *ε* is a vector of residuals. We transform all non‐binary variables by taking a natural logarithm to allow for non‐linear relationships in the data.

### Comparison with office location approaches

After we satisfy ourselves that IOPA provides an adequately valid empirical model of the WCN, we conduct tests for convergent validity of OLAs by comparing their network structure to IOPA. Messick (1995) proposes a test for convergent validity, which allows potentially valid measures to be validated by studying their correlations with other measures of the same concept, which are believed to be valid.

We compare IOPA network structures to five OLAs previously considered by Neal (2014) in the context of construct validity of empirical models of the WCN. They are distinct, albeit interrelated, and all of them rely on a firm–city affiliation matrix *F* representing the locations of APS firms' offices across cities as a primary data input.

Naïve (count) approach derives dyadic ties among cities simply as the product of their sizes, measured as the number of firms located in a given city.

(7) $$N=\left(F1\right){\left(F1\right)}^{{\;}^{\prime }}$$

where *N* is a weighted adjacency matrix with elements *n_ij_* equal to products of city sizes, and *F1* is a vector of city sizes, obtained by summing elements of a binary firm‐city affiliation matrix *F* for each row.

Taylor's (2001) IWCNM derives potential intercity business flows from input data on office locations across a set of cities. Number of firm overlaps for each city‐dyad, weighted by office size, serves as a proxy for intercity business flows.

(8) $$I=FF^{\prime }$$

where *I* is an adjacency matrix of intercity business flows and is derived as a product of firm‐city affiliation matrix *F* and its transpose *F′*. For *F* with elements *f_ij_* representing codified office sizes, we obtain *I* with elements *i_ij_* representing potential intercity business flows weighted by products of office sizes (IWCNM (weighted)). For *F* with elements *f_ij_* representing binary (0,1) dummies, indicating the presense of offices of firms in cities, we obtain *I* with elements *i_ij_* representing potential intercity business flows weighted by the number of overlapping firms for the given city‐dyad (IWCNM (binary)).

Normalized Bonacich (1972) approach instead considers the similarity of the populations of firms for each city‐dyad and is independent of nodal degree. In this sense, it is independent of city size, unlike the previous approaches.

(9) $$m_{\mathrm{ij}}=\frac{ad-\sqrt{\mathrm{abcd}}}{ad-bc};\;m_{\mathrm{ij}}=0.5\;\text{if}\;ad=bc$$

where *m_ij_* is a measure of firm overlap for city‐dyad *ij*, *a* is the number of overlapping firms for city‐dyad *ij*, *b* and *c* are the number of firms located only in cities *i (b)* or *j (c)* and *d* is the number of firms not located in either city *i* or *j*.

Finally, we also consider Neal's (2013) sorting model, based on identifying city‐dyads with statistically significant number of overlapping firms. Sorting model derives statistical significance by contrasting the observed number of overlapping firms for each city‐dyad to their empirical distribution obtained from a random sorting process detailed in equation (10) below.

(10) $$\mathrm{Pr}\left(rand_{\mathrm{ij}}\geq I_{\mathrm{ij}}\right)=\sum_{X=I_{\mathrm{ij}}}^{F}\frac{\left(\begin{array}{c} F\\ X \end{array}\right)\left(\begin{array}{c} F-X\\ I_{\mathrm{ii}}-X \end{array}\right)\left(\begin{array}{c} F-I_{\mathrm{ii}}\\ I_{\mathrm{jj}}-X \end{array}\right)}{\left(\begin{array}{c} F\\ I_{\mathrm{ii}} \end{array}\right)\left(\begin{array}{c} F\\ I_{\mathrm{jj}} \end{array}\right)}$$

(11) $$S_{\mathrm{ij}}=1\;if\;\mathrm{Pr}\left(rand_{\mathrm{ij}}\geq I_{\mathrm{ij}}\right)<\alpha ;\;S_{\mathrm{ij}}=0,\;\text{otherwise}$$

where *F* is the total number of firms sampled, *I_ii_* is the number of firms' offices located in a city *i* (proxy for capacity to host firms), *I_jj_* is the number of firms' offices located in a city *j*, X (also *I_ij_*) is the number of co‐located firms for given city‐dyad and *S_ij_* is a (0,1) binary variable indicating the presence of statistically significant network ties.

To facilitate a comparison among IOPA and OLAs, we use quadratic assignment procedure (QAP) correlation and regression techniques (Krackhardt 1988). QAP correlation coefficient ranges from −1 to 1 and can be interpreted as a measure of similarity for pairwise combinations of network structures. We obtain measures of statistical significance of these correlation coefficients using QAP permutation test based on 1,000 permutations of rows and columns in adjacency matrices.

We estimate a series of bivariate QAP regression models to compare the network structures of IOPA (reference model) and OLAs (tested models). We use the R^2^ from these models, a measure of model’s fit, to assess the convergent validity of each tested OLA. R^2^ varies from 0 (completely different network structure) to 1 (the same network structure) and can be consequently interpreted in this context as a (0,1) interval score of convergent validity, where 0 means completely invalid and 1 means completely valid.

### Data

To illustrate our approach empirically, we use data from Dealogic Equity Capital Market (ECM) and Debt Capital Market (DCM) databases, which represent detailed account of primary issues of equity and debt securities worldwide. They cover more than 100,000 initial public offerings, follow‐on offerings, convertibles and over 1 million deals involving debt securities in the period 2000–2015. We sample the top 500 bookrunner subsidiaries by total value of underwritten deals separately for ECM and DCM for each year from 2000 to 2015. We then hand collect data on the metropolitan statistical areas of their operational headquarters from a variety of sources including Bureau van Dijk Orbis, Nexis UK, Bloomberg and corporate websites. We sample all the syndicated deals that these banks were involved in, regardless of their role in each syndicate. This allowed us to cover between 96.65% and 99.96% of syndicated deals across the two products and period studied, totalling 161,114 syndicated deals.

For the purposes of comparing network structures derived from IOPA and OLAs, we construct a more detailed data set for 2015. We identify all 13,666 syndicated deals included in Dealogic ECM and DCM databases in 2015—2,539 equity securities and 11,247 debt securities issues featuring 1,398 underwriters in total. We then hand collect data on office locations of their subsidiaries, representing business units involved in underwriting these deals, from corporate websites, Bureau van Dijk Orbis, Nexis UK and Bloomberg. This yields a network of 2,192 offices of 1,398 firms located across an archipelago of 302 cities worldwide.

## Empirical evidence

**Figure 1:**
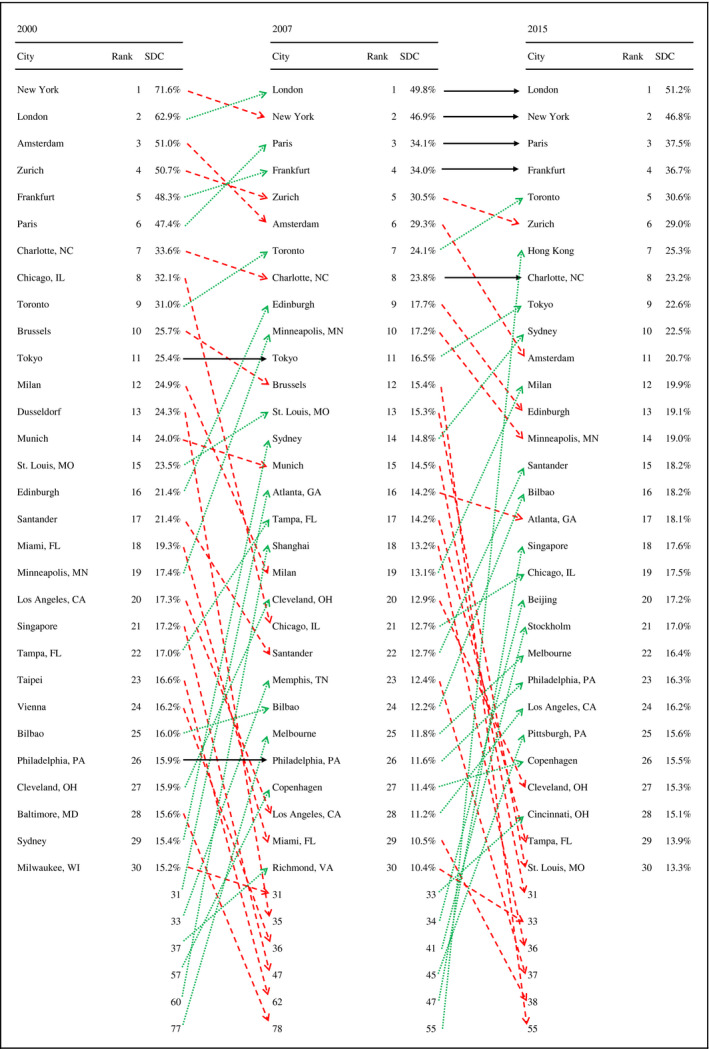
Ranking of cities by network centrality. Notes: SDC – Standardized group degree centrality. Source: Authors' analysis of Dealogic data.

Fig. 1 presents city degree centralities and their evolution between 2000 and 2015. Standardized group degree centralities shown in Fig. 1 can be interpreted as percentages of banks outside of each financial center (FC) that the banks within the given FC are connected to. In contrast to rankings and centrality scores based on OLAs (Taylor and Aranya 2008), both the rankings and centrality scores of cities based on directly observable syndicate co‐membership display more temporal and cross‐sectional variance, particularly so for cities of lower ranks. At the same time, the top of the ranking table is relatively stable over time, reflecting inertia in the development of leading global financial centers (Wójcik, Knight and Pažitka 2018). We observe a trend of declining network connectivity in the run‐up to the global financial crisis (GFC) and a partial recovery thereafter for well‐established FCs in developed economies, while those in the emerging markets, with China in the lead, have strengthened their connectivity in the post‐GFC period. Australia is an exception to this pattern, with both Sydney and Melbourne increasing their network centrality steadily from 2000 to 2015.

**Figure 2:**
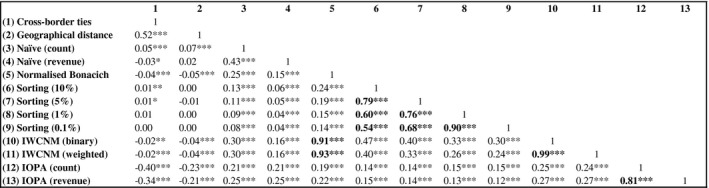
Correlation matrix. Notes: Statistically significant at ***1%, **5% and *10% level. Correlation coefficients are obtained using quadratic assignment procedure (QAP) correlation (Krackhardt 1988). Statistical significance of QAP correlation coefficients is assessed using QAP permutation test based on 1,000 permutations of rows and columns in adjacency matrices. Source: Authors' calculations based on Dealogic data.

We begin our comparison of urban network structures by examining QAP correlations, which we interpret as measures of similarity among network structures. Fig. 2 presents a QAP correlation matrix. The first striking result is that all the network structures produced by OLAs are either uncorrelated or extremely weakly correlated with geographical distance and political borders. The lack of any meaningful negative correlation between these proxies for barriers to interactions and the projected intercity business flows derived from OLAs calls into question the structural validity of these approaches. In contrast, network structures produced by IOPA are negatively correlated with both geographical distance among cities and political borders, as expected. Naïve (revenue) variable, a proxy for interaction potential among cities, is positively correlated with all OLAs and IOPA network structures, although it is noteworthy that network structures produced by the sorting model are very weakly correlated with this variable. Network structures derived using IOPA are only very weakly correlated with those of OLAs. Taylor's (2001) IWCNM displays the highest correlation with IOPA, while Neal's (2013) sorting model is least correlated with IOPA.

Table 1 summarizes the results of our tests of structural validity. The naïve (count) network structure is not statistically significantly related to either geographical distance or political borders, which is not surprising given its functional form. Normalized Bonacich, IWCNM (binary) and IWCNM (weighted) are statistically significantly related to geographical distance and dyadic products of city sizes (naïve (revenue)), although they are not related to political borders. The sorting (10%) model has a positive coefficient estimate on cross‐border ties, implying more flows across political borders than within them and is not related to geographical distance. This means that neither of the five OLAs passes this test of structural validity. The IOPA (count) and IOPA (revenue) network structures are negatively and statistically significantly related to both geographical distance and political borders. They are also positively and statistically significantly related to naïve (revenue), a proxy for interaction potential among cities. The R^2^ of these models is 0.195 (IOPA (count)) and 0.177 (IOPA (revenue)), meaning that almost 20% of variance in these network structures can be explained by these three factors. We therefore conclude that IOPA passes this test of structural validity.

**Table 1:** Tests of structural validity

	Naïve (count)	Normalized Bonacich	IWCNM (binary)	IWCNM (weighted)	Sorting (10%)	IOPA (count)	IOPA (revenue)
	[1]	[2]	[3]	[4]	[5]	[6]	[7]
				$\hat{\beta }$ ***			
				(*P*‐value)			
Cross‐border ties	0.1216	‐0.0023	0.0001	0.0014	0.0011*	−0.3230***	−1.5492***
	(0.13)	(0.28)	(0.99)	(0.88)	(0.05)	(0.00)	(0.00)
Geographical distance	0.0621	−0.0039***	−0.0062***	−0.0162***	−0.0004	−0.0189***	−0.1205***
	(0.15)	(0.00)	(0.01)	(0.00)	(0.13)	(0.00)	(0.00)
Naïve (revenue)	0.0397***	0.0009***	0.0016***	0.0041***	0.0001***	0.0054***	0.0379***
	(0.00)	(0.00)	(0.00)	(0.00)	(0.00)	(0.00)	(0.00)
Adjusted *R* ^2^	0.190	0.024	0.027	0.028	0.004	0.195	0.177

Notes:

All variables are included in the form of adjacency matrices with dimensions 302 × 302, yielding 91,204 observations. Coefficient estimates are estimated using quadratic assignment procedure (QAP) regression (Krackhardt 1988). Standard errors and *P*‐values of these coefficient estimates are obtained using QAP permutation test based on 1,000 permutations of rows and columns in adjacency matrices. Statistically significant at ***1% level, **5% level and *10% level.

Having satisfied ourselves with the above tests of structural validity of IOPA, we now proceed to estimate a series of bivariate models, which compare the network structure produced by IOPA with OLAs (Table 2). The primary criterion in tests for convergent validity of OLAs is their explanatory power (R^2^), which can be interpreted as a (0,1) interval score of convergent validity. Consequently, if a modeling approach has a R^2^ close to 1, we conclude that its network structure is highly consistent with observable intercity business flows, represented by IOPA, and therefore passes the test of convergent validity. If its R^2^ is close to zero, we conclude that the given approach yields a network structure inconsistent with observable intercity business flows and does not pass the test of convergent validity. The R^2^ in Table 2 ranges from 0.020 for the sorting (10%) to 0.060 for IWCNM (binary) model, when we use IOPA (count) as a dependent variable. Similarly, for the IOPA (revenue) dependent variable, it ranges from 0.022 for sorting (10%) to 0.075 for IWCNM (binary) model. This therefore implies that the OLAs considered here are only weakly associated with observable intercity business flows and they do not meet the criteria for convergent validity in this test.

**Table 2:** Comparison of IOPA with OLAs' network structures

	IOPA (count)					IOPA (revenue)				
	[1]	[2]	[3]	[4]	[5]	[6]	[7]	[8]	[9]	[10]
						$\hat{\beta }$ ***				
						(*P*‐value)				
Naïve (count)	0.0624***					0.4137***				
	(0.00)					(0.00)				
Normalized Bonacich		0.8871***					5.7889***			
		(0.00)					(0.00)			
IWCNM (binary)			0.6633***					4.1963***		
			(0.00)					(0.00)		
IWCNM (weighted)				0.2599***					1.6620***	
				(0.00)					(0.00)	
Sorting (10%)					1.9252***					11.5200***
					(0.00)					(0.00)
Adjusted *R* ^2^	0.044	0.037	0.060	0.056	0.020	0.060	0.049	0.075	0.072	0.022

Notes:

All variables are included in the form of adjacency matrices with dimensions 302 × 302, yielding 91,204 observations. Models 1–5 feature IOPA (count) adjacency matrix and models 6–10 feature IOPA (revenue) adjacency matrix as their dependent variable. Coefficient estimates are estimated using quadratic assignment procedure (QAP) regression (Krackhardt 1988). Standard errors and *P*‐values of these coefficient estimates are obtained using QAP permutation test based on 1,000 permutations of rows and columns in adjacency matrices. Statistically significant at ***1% level, **5% level and *10% level.

## Conclusions

The first objective of this article was to examine the content, construct and structural validity of OLAs as empirical models of the WCN (Taylor and Derudder 2016). By doing so, we are hoping to advance the dialogue on the appropriateness of the IWCNM and other OLAs for indirectly measuring intercity business flows and to further motivate research focusing on developing, formalizing and validating empirical models of the WCN. Our examination of the construct validity of OLAs raises several concerns, which make us question the appropriateness of this class of methods for measuring intercity business flows. First, OLAs presuppose that a material support for business flows, offices of firms, relate in a very specific and knowable manner to intercity business flows. Second, projections of intercity business flows obtained using OLAs assume that the propensity for interaction among a pair of offices of a given relative importance does not vary across firms or industries. Third, OLAs' projections of intercity business flows are based solely on intra‐firm links among offices located in different cities and interfirm interactions are excluded by construction. Consequently, it concerns us that important sources of variation in intercity business flows are omitted and there is also a source of spurious variation in network structures projected by OLAs.

The empirical evidence presented in this article corroborates our concerns and shows that projections of intercity business flows obtained using OLAs have a structure that is at odds with the current understanding of propensity to interaction at distance in urban and economic geography (Polèse 2010) as well as international economics (Moshirian, Li, and Sim 2005). We find that projections of intercity business flows obtained using some OLAs do not decline with geographical distance and most OLAs yield projections that are unaffected by national borders. None of the OLAs considered here yield projections of intercity business flows that are statistically significantly related to both geographical distance among cities and the presence of national borders, which we understand as an empirical red flag.

The second objective of this article was to develop, formalize and validate an empirical model of the WCN, based on directly observable relational ties among APS firms, formed through inter‐organizational projects, which are directly related to co‐production of complex services. IOPA is rooted in the co‐production of APS as a central construct in global city theory (Sassen 2001) and aims to measure intercity business flows that form the WCN (Taylor and Derudder 2016). We introduce IOPA, demonstrate it empirically, argue for its content and construct validity and offer empirical evidence for its structural validity (Cronbach and Meehl 1955; Messick 1995). We argue that network projections of the WCN derived using IOPA capture intercity business flows more directly and accurately than those derived using OLAs. IOPA can therefore be a viable alternative to OLAs and can in fact be used to assess their convergent validity, a test that could not be performed previously due to the lack of adequately valid reference models.

Our third objective was to use IOPA as a reference model for testing the convergent validity of OLAs. We show that OLAs yield network structures that are only weakly related to the observed structure of intercity business flows derived using IOPA. This evidence combined with the identified problems of construct validity and failed empirical tests of structural validity of OLAs lead us to believe that although these approaches help investigate office location networks, their ability to offer valid measures of intercity business flows, the centerpiece of the WCN construct, is limited. Indeed, this issue afflicting OLAs may get worse over time as firms increasingly use modes of product and service delivery that bypass traditional office networks, for example digital platform and infrastructure, and travel (Castells 1996; Knight and Wójcik 2017).

Although our empirical analysis is limited to investment banking, IOPA could be extended to a much broader set of APS and other industries. Parallels can be drawn between underwriting syndicates and other types of inter‐organizational projects, such as syndicated loans, syndication of venture capital general partners, co‐counseling of legal cases by law firms, collaboration of marketing firms on advertising campaigns or multiple management consultancy firms advising a client on an acquisition strategy (Knight and Wójcik 2017). We therefore see our approach as being broadly applicable to a wide range of inter‐organizational projects among APS firms related to co‐production of complex services and a powerful tool for studying urban networks.

Our operationalization of the IOPA is naturally subject to several limitations. Firstly, although we use relational data, which we argue represent intercity business flows, they do so in a crude manner. Using the number of ties among firms formed through inter‐organizational projects may not give a precise estimate of the flows of information, money or people. This can be alleviated by weighting these transactions by revenue earned from them, but only partially so. Secondly, we have shown that data on capital market transactions is plentiful and comprehensive global data sets can be built within the bounds of resources typically available to researchers. However, it remains a question how difficult it would be to extend this analysis to a broader set of APS sectors, such as those more conventionally covered in the WCN research (Taylor and Derudder 2016). Thirdly, inter‐organizational projects are only one of many ways in which APS firms may be connected. Ideally, a more comprehensive approach would aim to account for the multitude of business flows and network interactions that exist among organizations (Wójcik 2018).

Despite our critique of IWCNM and OLAs as empirical models of the WCN, we do not suggest that there is no use for office location data. In fact, the many data sets covering office locations of APS firms currently available can be fruitfully applied to studying spatial organization of firms and modeling location choices, thus shedding light on the characteristics of cities that make them attractive to APS firms. Our results serve as an opportunity to open new research domains for global and world city scholarship that is more attuned to the finer details of intercity business flows and strategic firm activities that our model captures. There are opportunities for future research to extend this work by identifying new data sets and contexts, while utilizing IOPA to study different APS sectors, time periods and alternative types of inter‐organizational projects. Data should not be as major a limitation, as it may have been in the past. While Dealogic data used in this article is proprietary, we understand that similar data sets could be built using a more widely available Thomson Reuters Eikon database among others. We plan to and would like to encourage scholars to look for other sources of data that can be validly linked to intercity business flows. In these pursuits, our advice is to always verify the content, construct and structural validity of empirical measures under consideration (Cronbach and Meehl 1955; Messick 1995).

## Notes

Neal (2014) uses the term “measurement validity,” instead of construct validity, although it is apparent that he also interrogates the link between empirical measures and the theoretical concepts they are supposed to represent.

City size is measured in this context by the aggregate revenue of sampled firms located in a city.
